# Obesity-Associated Myeloid-Derived Suppressor Cells Promote Apoptosis of Tumor-Infiltrating CD8 T Cells and Immunotherapy Resistance in Breast Cancer

**DOI:** 10.3389/fimmu.2020.590794

**Published:** 2020-10-06

**Authors:** Justin T. Gibson, Rachael M. Orlandella, William J. Turbitt, Michael Behring, Upender Manne, Robert E. Sorge, Lyse A. Norian

**Affiliations:** ^1^ Graduate Biomedical Sciences, University of Alabama at Birmingham, Birmingham, AL, United States; ^2^ Department of Nutrition Sciences, University of Alabama at Birmingham, Birmingham, AL, United States; ^3^ Department of Pathology, University of Alabama at Birmingham, Birmingham, AL, United States; ^4^ O’Neal Comprehensive Cancer Center, University of Alabama at Birmingham, Birmingham, AL, United States; ^5^ Department of Psychology, University of Alabama at Birmingham, Birmingham, AL, United States; ^6^ Nutrition Obesity Research Center, University of Alabama at Birmingham, Birmingham, AL, United States

**Keywords:** ****myeloid-derived suppressor cells (MDSCs), obesity, immunotherapy resistance, breast cancer, tumor-infiltrating lymphocyte apoptosis, Fas ligand (FasL)

## Abstract

Nearly 70% of adults in the US are currently overweight or obese. Despite such high prevalence, the impact of obesity on antitumor immunity and immunotherapy outcomes remains incompletely understood, particularly in patients with breast cancer. Here, we addressed these gaps in knowledge using two murine models of breast cancer combined with diet-induced obesity. We report that obesity increases CXCL1 concentrations in the mammary tumor microenvironment, driving CXCR2-mediated chemotaxis and accumulation of granulocytic myeloid-derived suppressor cells (G-MDSCs) expressing Fas ligand (FasL). Obesity simultaneously promotes hyperactivation of CD8 tumor-infiltrating lymphocytes (TILs), as evidenced by increased expression of CD44, PD-1, Ki-67, IFNγ, and the death receptor Fas. Accordingly, G-MDSCs induce Fas/FasL-mediated apoptosis of CD8 T cells *ex vivo* and *in vivo*. These changes promote immunotherapy resistance in obese mice. Disruption of CXCR2-mediated G-MDSC chemotaxis in obese mice is sufficient to limit intratumoral G-MDSC accumulation and improve immunotherapy outcomes. The translational relevance of our findings is demonstrated by transcriptomic analyses of human breast tumor tissues, which reveal positive associations between *CXCL1* expression and body mass index, poor survival, and a MDSC gene signature. Further, this MDSC gene signature is positively associated with *FASLG* expression. Thus, we have identified a pathway wherein obesity leads to increased intratumoral CXCL1 concentrations, which promotes CXCR2-mediated accumulation of FasL^+^ G-MDSCs, resulting in heightened CD8 TIL apoptosis and immunotherapy resistance. Disruption of this pathway may improve immunotherapy outcomes in patients with breast cancer and obesity.

## Introduction

Obesity is a major health epidemic in the US, affecting nearly 40% of adults (1). Excess body weight increases the incidence of 13 types of cancer (2), promotes disease progression (3, 4), impairs antitumor immunity (5, 6), promotes resistance to targeted and chemotherapies (7, 8), and worsens survival for cancer patients (9, 10), especially those with breast cancer. However, the impact of obesity on immunotherapy outcomes in patients with breast cancer is unknown.

In 2019, the FDA granted accelerated approval for the treatment of select triple negative breast cancer patients with an immunotherapy/chemotherapy combination based on results reported from the phase III IMpassion130 clinical trial (11). However, despite immunotherapeutics dramatically improving outcomes for a subset of patients with cancer, many still do not respond. As such, research efforts are concentrated on identifying and targeting factors underlying immunotherapeutic resistance. Obesity may be one such factor. Recent findings from a phase I clinical trial of patients with breast cancer treated with atezolizumab (anti-PD-L1) indicate that elevated baseline plasma levels of interleukin 6 (IL-6) and C-reactive protein (CRP) are associated with reduced progression free and overall survival (12). As both IL-6 and CRP are biomarkers of obesity-associated inflammation, this raises the possibility that obesity may impair immunotherapeutic efficacy in patients with breast cancer. In support of this idea, obesity was previously shown to be associated with impaired responses to targeted and chemotherapies in patients with breast cancer (7, 8). However, given the short duration of its clinical use, the impact of obesity on immunotherapy outcomes in patients with breast cancer has not yet been investigated.

Molecular markers of obesity (e.g. IL-6, CRP, leptin, interleukin 1 beta [IL-1β]) increase the generation of myeloid-derived suppressor cells (MDSCs) (13–16). MDSCs are a heterogeneous immunosuppressive population of cells that function to inhibit protective antitumor immunity to promote tumor growth and progression. To date, only one study has investigated the impact of obesity-associated MDSCs in mediating mammary tumor growth (14). However, this study did not assess the impact of MDSCs on response to immunotherapy. Given that intratumoral accumulation of MDSCs has been identified as a mechanism of immunotherapy resistance (17, 18), we asked if obesity would enhance MDSC-mediated immunotherapy resistance in a preclinical model of breast cancer.

## Materials and Methods

### Research Resource Identifiers

Research Resource Identifiers (RRIDs) and other detailed identifier information (vendor, clone, catalog number etc.) for antibodies and key reagents used in this study can be found in this text and **Supplementary Table 1**.

### Murine Models of Obesity

Wildtype C57BL/6 female mice were purchased from Charles River at 7–8 weeks of age. Following one week of acclimation, animals were randomized to a NIH31 formulated low-fat diet (LFD; 14% kcal from fat, LabDiet), standard American diet (SAD; 36% kcal from fat, Envigo), or high-fat diet (HFD; 60% kcal from fat, Research Diets) for 16 weeks, thereby generating lean (LFD) or diet-induced obese (SAD and HFD) mice. All animal experiments were conducted as approved by the Animal Resources Program and Institutional Animal Care and Use Committee at the University of Alabama at Birmingham (UAB).

### In Vivo Tumor Modeling

The E0771 murine mammary tumor cell line was purchased from CH3 BioSystems (RRID: CVCL_GR23) and engineered to express firefly luciferase (E0771-fluc) *via* transduction with lentiviral particles (Kerafast) according to the manufacturer’s protocol. Cells were maintained in RPMI1640 complete media supplemented with 10% FBS, 10mM HEPES, 100 units/ml Penicillin, and 100 µg/ml Streptomycin. The Py8119 murine mammary tumor cell line was purchased from ATCC (RRID : CVCL_AQ09) and cells were maintained in F-12K complete media supplemented with 5% FBS. Neither cell line was authenticated after purchase and both were determined to be free of mycoplasma contamination.

Mice were injected with 2.5e5 E0771-fluc or 1e6 Py8119 cells subcutaneously into mammary fat pad number four. Tumor outgrowth was monitored using a dial caliper and bioluminescence imaging. To measure E0771-fluc tumor bioluminescence, tumor-bearing mice were injected intraperitoneally with 1mg of luciferin, anesthetized, and imaged using an IVIS Lumina III imaging system and the Living Image Software Version 4.7.2 (Perkin Elmer).

### NanoString and Gene Ontology Analysis of Tumor Immune Microenvironment

At day 28 experimental endpoint, E0771-fluc tumors were excised and a portion was preserved in RNAlater. RNA was batch isolated and submitted to the UAB nanoString facility for interrogation using the nCounter PanCancer Immune Profiling Panel (XT-CSO-MIP1-12) according to the manufacturer’s instructions. Data analysis was performed using nCounter Analysis Software (Version 4.0) with the Advanced Analysis (Version 2.0) plugin. Differentially expressed genes were identified as those with a p value < 0.05 and fold change > ± 1.5. Due to the exploratory nature of our study and intent for validation, we used raw p values and did not adjust p values for multiple comparisons.

The nanoString tumor immunogenetic profiling dataset expression matrix from n=72 subjects with triple negative breast cancer (TNBC) was obtained from a previously published manuscript (17). Body mass index (BMI) information for this cohort of subjects was kindly provided by Dr. Xiang “Shawn” Zhang, Baylor College of Medicine. Of the 72 patient tissues evaluated, 4 were excluded from analysis due to lack of BMI data, being underweight, or issues with sample quality control. The final cohort for analysis consisted of n=68 samples. Linear regression analyses were performed for BMI versus log2 expression of each of the 730 genes. Due to the exploratory nature of the study, those with a p<0.1 were investigated further. Of the 730 genes, 73 were associated with BMI; 8 positively and 65 negatively.

From our murine study, differentially expressed genes were separated into groups of upregulated or downregulated genes and subjected to Gene Ontology enrichment analysis using the PANTHER classification system to identify associated biological processes (http://geneontology.org/) (19). This was also done for the clinical analyses using genes positively associated with BMI, identified as described above.

### Flow Cytometry

Spleen and tumor tissues were excised and mechanically homogenized using the Miltenyi gentleMACS Dissociator, followed by enzymatic digestion in 5 µg/ml Liberase TM and 37.5 µg/ml DNase I at 37°C with gentle agitation for 15 or 30 min, respectively. Resulting homogenates were filtered (70um) to obtain single cell suspensions. Cells were stained with fixable Zombie Aqua viability dye, Fc blocked, and incubated with fluorophore-conjugated antibodies to surface markers. Evaluation of intracellular markers was conducted using BD’s Cytofix/Cytoperm kit according to manufacturer’s protocol. For evaluation of T cell cytokine production, single cell tumor homogenates were stimulated with anti-CD3 (8 µg/ml) and anti-CD28 (10 µg/ml) for 4 h with the addition of GolgiPlug during the last 2 h. Cells were then harvested and stained as indicated above for surface and intracellular markers. Apoptosis was evaluated using the BD Annexin V Apoptosis Detection Kit according to manufacturer’s protocol.

Samples were analyzed on an Attune NxT cytometer after compensation using BD Comp Beads. Analyses were conducted using FlowJo (Version 10) and all gates were objectively drawn based on fluorescence minus one (FMO) controls for the tissue of interest. Detailed information for the antibodies used in this study is provided in **Supplementary Table 1**.

### Quantification of Soluble Proteins

Tumors were excised and placed in Dulbecco’s Phosphate Buffered Saline (DPBS; 0.2g tissue per 1 ml DPBS) followed by mechanical homogenization using a Miltenyi gentleMACS Dissociator. Tumor homogenates were centrifuged and supernatants were collected and stored for batch analysis of soluble proteins using the Bio-Plex Multiplex Immunoassay (Bio-Rad) according to the manufacturer’s protocols.

### In Vivo Anti-CD8β Antibody-Mediated CD8 T Cell Depletion

On days 1, 3, 5, 7, 14, 21, and 27 post tumor challenge, lean and DIO animals received an intraperitoneal injection containing 100 µg of anti-CD8β or isotype control antibody (Bio X Cell). Animals were euthanized on day 28 post tumor challenge, at which time tumor tissues were excised and CD8 TILs were evaluated by flow cytometry.

### In Vivo Anti-Gr-1 Antibody-Mediated MDSC Depletion

On day 19 post tumor challenge, DIO animals received an intratumoral injection containing 200 µg of anti-Gr-1 or isotype control antibody (Bio X Cell). On days 20, 21, and 22 post tumor challenge, animals received a follow-up injection containing 100 µg of their respective antibody. Animals were euthanized on day 23 post tumor challenge, at which time tumor tissues were excised and infiltrating leukocytes were evaluated by flow cytometry.

### G-MDSC Induction of Apoptosis

CD8^+^ T cells were positively selected from spleens of naive 8- to 12-week-old C57BL/6 female mice using Miltenyi MACS Enrichment according to the manufacturer’s protocol. Enriched CD8^+^ T cells were activated *in vitro* for 72 h using 1 µg/ml anti-CD3 and 1 µg/ml anti-CD28 to induce Fas expression. Tumor-infiltrating G-MDSCs from DIO mice were sort-purified on a BD FACSMelody after being pre-enriched using anti-CD11b MACS beads from Miltenyi, as per the manufacturer’s protocol. Prior to co-culture, purified G-MDSCs were incubated in the presence or absence of 50 µg/ml anti-FasL neutralizing antibody for 1 h. *In vitro* activated CD8^+^ T cells were then co-cultured with G-MDSCs in the continued presence or absence of anti-FasL for 24 h. Cells were harvested and stained for flow cytometry to evaluate CD8 T cell apoptosis as indicated above.

### Immunotherapy

Mice with established tumors within a 25–100 mm^2^ range (~50 mm^2^ on average) were randomized to receive saline or immunotherapy. Immunotherapy consisted of 1x10^9^ PFU of adenovirus (Ad) encoding murine TNF-related apoptosis inducing ligand (TRAIL; AdT) and 50 µg of the TLR9 agonist CpG 1826 oligodeoxynucleotide (AdT+CpG) (20). Saline or AdT+CpG were administered in 50uL volumes *via* intratumoral injection.

Disruption of CXCR2 signaling was accomplished using the CXCR2 antagonist SB 225002 (Tocris Bioscience). Beginning 2 days prior to tumor challenge and every 2 days after until day 26, 5 mg/kg of CXCR2a was administered intraperitoneally to lean and DIO animals.

### Clinical Outcomes

Kaplan-Meier Plotter (KM Plotter; https://kmplot.com/) was used to evaluate the association of *CXCL1* expression and overall survival in systemically untreated patients with basal breast cancer (21). The following parameters were used for analysis with regard to *CXCL1*: auto select best cutoff, overall survival, intrinsic subtype: basal, and systemically untreated patients. Log rank p value and hazard ratio (HR) were calculated as part of the analysis.

### Clinical Transcriptomic Analyses

Transcriptomic data were downloaded from The Cancer Genome Atlas (TCGA) using the curatedTCGAData package (https://bioconductor.org/packages/release/data/experiment/html/curatedTCGAData.html). The most recent version of TCGA RNASeq data (title “BRCA_RNASeq2GeneNorm-20160128”) was used for analysis. To approximate expression values, RNASeq2 count data were log2 transformed and the mean expression of a previously published multi-gene MDSC signature (22) was calculated. The MDSC expression score was then separated into tertiles and compared to log2 expression values for genes of interest in tumor tissues from n=88 patients with basal breast cancer.

### Statistical Analysis

Statistical analyses were performed using GraphPad Prism Version 7.0. Gaussian distribution was assessed using the Shapiro-Wilk normality test. Comparisons of two groups was accomplished using non-parametric Mann-Whitney U tests or parametric two-tailed unpaired t-tests as appropriate. Comparisons of three groups was accomplished using non-parametric Kruskal-Wallis tests with Dunn’s multiple comparisons tests or parametric one-way ANOVAs with Tukey’s or Dunnet’s multiple comparisons tests as appropriate. Analysis of four groups was carried out using non-parametric Kruskal-Wallis tests or parametric one-way ANOVAs with post-hoc pairwise comparisons of group means of interest using non-parametric Mann-Whitney U tests, parametric two-tailed unpaired t-tests, or Tukey’s multiple comparisons test, as appropriate. Differences in tumor growth over time were assessed using repeated measures two-way ANOVAs with Tukey’s multiple comparisons test. Associations between clinical gene expression and BMI were evaluated using linear regression analyses. Statistical differences are denoted throughout as *p < 0.05, **p < 0.01, ***p < 0.001, ****p < 0.0001. Non-significant (ns) p values ≤ 0.15 are indicated throughout.

## Results

### Obesity Promotes Mammary Tumor Growth and Remodeling of the Tumor Immune Microenvironment

**Figure 1:**
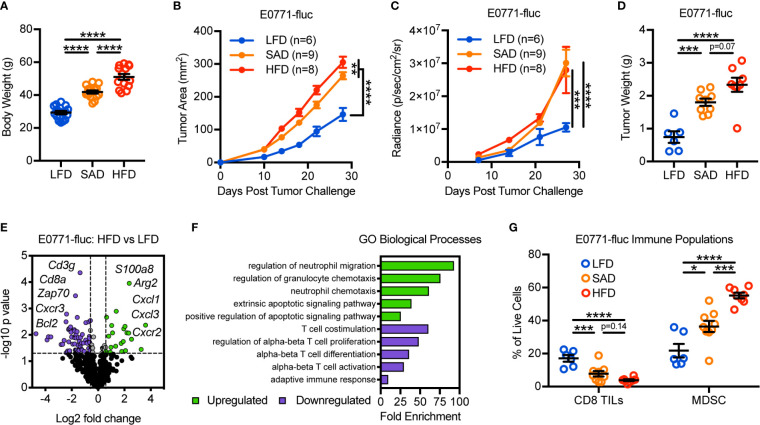
Diet-induced obesity promotes mammary tumor growth and remodeling of the tumor immune microenvironment. **(A)** Body weight of animals 16 weeks after randomization to low-fat diet (LFD; 14% kcal from fat), standard American diet (SAD; 36% kcal from fat), or high-fat diet (HFD; 60% kcal from fat). Following diet randomization, animals were challenged with E0771-fluc mammary tumor cells and tumor growth monitored *via* **(B)** caliper measurements, **(C)** bioluminescent imaging of firefly luciferase expressing E0771 tumor cells (E0771-fluc), and **(D)** excised tumor weights. **(E)** Volcano plot showing differentially expressed (DE) genes from tumors of HFD-fed animals vs LFD-fed animals. Dotted lines indicate p = 0.05 and fold change = ±1.5. **(F)** Gene Ontology (GO) enrichment analysis for biological processes associated with downregulated (purple) and upregulated (green) DE genes from **(E)**. **(G)** Flow cytometric analysis of day 28 excised mammary tumors for CD3^+^CD8^+^ tumor-infiltrating lymphocytes and CD11b^+^Gr-1^+^ MDSCs. Data are pooled from multiple independent experiments and presented as means ± SEM. Statistical differences were calculated using parametric one-way ANOVAs or repeated measures two-way ANOVAs, both with Tukey’s multiple comparisons tests (*p < 0.05, **p < 0.01, ***p < 0.001, ****p < 0.0001).

To evaluate the impact of obesity on mammary tumor growth, we utilized two models of diet-induced obesity (DIO) wherein animals were randomized to either a standard American diet (SAD; 36% kcal from fat) or high-fat diet (HFD; 60% kcal from fat) for 16 weeks. Low-fat diet-fed (LFD; 14% kcal from fat) animals served as age-matched lean controls. SAD- and HFD-feeding led to significantly increased body weights relative to LFD-fed lean controls, with HFD-fed animals having further increases in body weights relative to SAD-fed animals (**Figure 1A**). Lean and DIO mice were then challenged with E0771 mammary tumor cells expressing firefly luciferase (E0771-fluc) and tumor growth was monitored *via* caliper measurements, bioluminescent imaging, and excised tumor weights. All methods indicated that both SAD- and HFD-induced obesity promote accelerated mammary tumor growth (**Figures 1B–D**).

We next asked how DIO impacted the composition of the tumor immune microenvironment. To this end, we evaluated the immunogenetic profile of tumors from LFD- and HFD-fed animals using nanoString to screen for genes of interest. Doing so revealed differential expression (DE) of 86 of the 730 genes surveyed (**Figure 1E** and **Supplementary Table 2**). Gene Ontology (GO) assessment of biological processes involved with downregulated genes largely identified T cell alterations, whereas upregulated genes were associated with neutrophil/granulocyte trafficking and apoptotic signaling (**Figure 1F**). Flow cytometric analysis confirmed obesity-associated reductions in CD8 tumor-infiltrating lymphocytes (TILs) and increases in myeloid-derived suppressor cells (MDSCs), a subset of which are phenotypically synonymous with tumor-associated neutrophils (**Figure 1G**). Prior studies have shown that these immunologic alterations are associated with negative clinical outcomes in patients with breast cancer, independent of obesity status (23, 24). Most notably, as body weights increased (LFD<SAD<HFD), there were corresponding increases in tumor burden and MDSC abundance, whereas CD8 TIL abundance decreased. Similar obesity-associated alterations to tumor growth and leukocyte composition were also seen in the Py8119 mammary tumor model, indicating that these results were not specific to a single tumor model (**Supplementary Figure 1**).

We next wanted to explore whether a causal relationship existed between the abundance of CD8 TILs and mammary tumor growth in LFD-fed lean and HFD-fed DIO mice. Following depletion of CD8 T cells, lean mice experienced accelerated tumor growth as expected, demonstrating the critical role of CD8 TILs in mediating antitumor immunity in the E0771-fluc tumor model (**Supplementary Figures 2A, B** **)**. Unsurprisingly, depletion of CD8 T cells in DIO mice did not impact mammary tumor growth (**Supplementary Figure 2A**). Quantification of CD8 TILs suggested that this was likely due to the already extremely low levels of CD8 TILs present in the obese tumor microenvironment (**Supplementary Figure 2B**). Thus these data suggested that obesity-associated reductions in CD8 TIL abundance impair antitumor immunity.

### Obesity Promotes CD8 TIL Hyperactivation and Apoptosis in the Mammary Tumor Microenvironment

**Figure 2:**
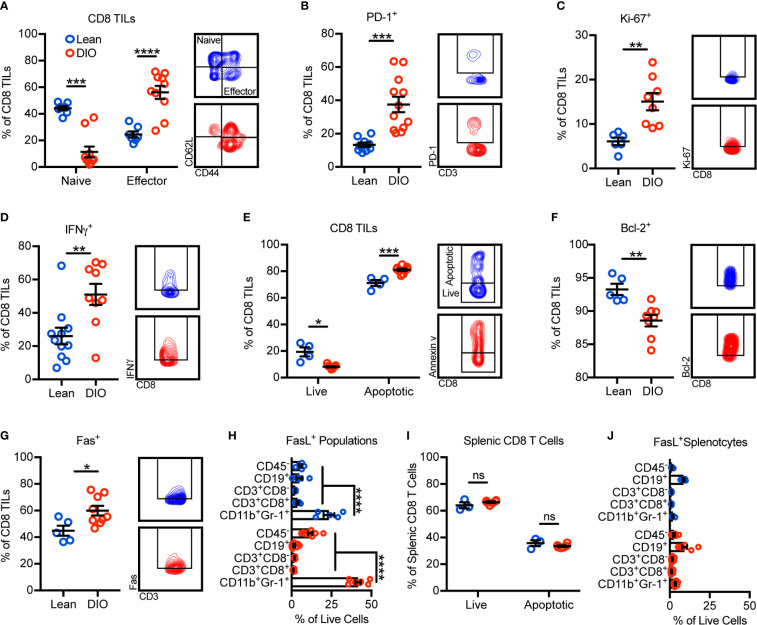
Obesity promotes CD8 TIL hyperactivation and apoptosis in the mammary tumor microenvironment. Flow cytometric analysis of day 28 excised E0771-fluc mammary tumors for **(A)** naive (CD44^-^CD62L^+^) and effector (CD44^+^CD62L^-^) CD8 TILs. Expression of **(B)** PD-1, **(C)** Ki-67, and **(D)** IFNγ by CD8 TILs. **(E)** Quantification of live (annexin V^-^) and apoptotic (annexin V^+^) CD8 TILs. Expression of **(F)** Bcl-2 and **(G)** Fas by CD8 TILs. **(H)** Quantification of FasL^+^ tumor infiltrating populations. **(I)** Quantification of live (annexin V^-^) and apoptotic (annexin V^+^) splenic CD8 T cells and **(J)** FasL^+^ splenic populations. **(A–D, F)** Data are pooled from multiple independent experiments or **(E, G–J)** are from a single representative experiment and presented as means ± SEM. Statistical differences were calculated using parametric t-tests or non-parametric Mann-Whitney U tests as appropriate and parametric one-way ANOVAs with Dunnet’s multiple comparisons tests (*p < 0.05, **p < 0.01, ***p < 0.001, ****p < 0.0001, ns = non-significant).

Given that obesity was associated with reductions in total CD8 TILs, we further sought to identify potential alterations in their phenotype and mechanisms underlying their reduction. Surprisingly, the frequency of CD8 TILs with an effector phenotype increased with obesity while naive CD8 TILs decreased (**Figure 2A**). Accordingly, increases were seen in the percentages of PD-1^+^, Ki-67^+^, and IFNγ^+^ CD8 TILs, consistent with increased antigen engagement, proliferation, and cytokine production with obesity (**Figures 2B–D**). Notably, regardless of phenotype, total effector and naive CD8 TIL subsets and intratumoral IFNγ concentration were decreased with obesity (**Supplementary Figure 3**). Our immunogenetic profiling results indicated that obesity was associated with increased apoptotic signaling (**Figure 1F**). Thus, we next asked if obesity-associated TIL hyperactivation promoted apoptosis, causing decreased CD8 TIL abundance (**Figure 1G**). Annexin V staining revealed that obesity was indeed associated with increased percentages of apoptotic CD8 TILs (**Figure 2E**). Further, DIO animals had reduced percentages of CD8 TILs expressing antiapoptotic Bcl-2 (**Figure 2F**) and increased percentages of CD8 TILs expressing the death receptor Fas (**Figure 2G**). These results corroborated our immunogenetic profiling results (**Figures 1E, F**) and indicated that CD8 TILs were hyperactivated and poised for apoptosis with obesity.

Having established that a higher percentage of CD8 TILs from DIO animals were expressing Fas and undergoing apoptosis, we next asked what sources of FasL existed within the tumor microenvironment that could potentially promote Fas/FasL-mediated apoptosis of CD8 TILs. In both lean and DIO animals, CD11b^+^Gr-1^+^ MDSCs were the largest contributors of FasL expression (**Figure 2H**). Notably, obesity did not impact the apoptotic phenotype of splenic CD8 T cells where FasL^+^ MDSCs were minimally abundant (**Figures 2I, J**). These results suggested that the abundance of FasL^+^ MDSCs was associated with CD8 TIL apoptosis.

### Obesity Promotes the Recruitment and Accumulation of FasL^+^ G-MDSCs Which Induce Fas/FasL-Mediated Apoptosis of Hyperactivated CD8 TILs

**Figure 3:**
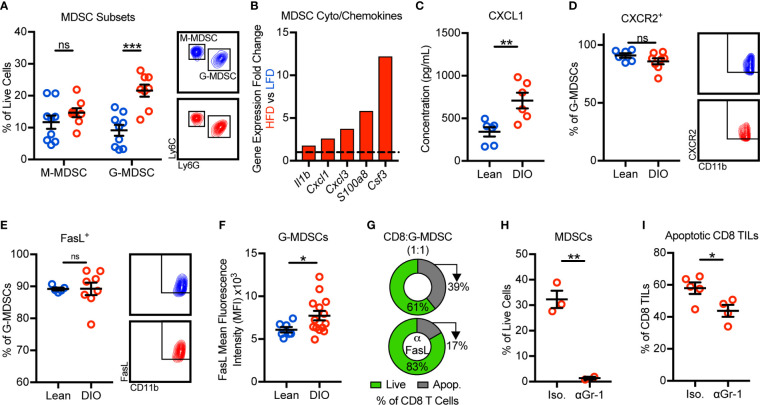
Obesity promotes the recruitment and accumulation of FasL^+^ granulocytic myeloid-derived suppressor cells (G-MDSCs) which induce Fas/FasL-mediated apoptosis of CD8 TILs. Flow cytometric analysis of day 28 excised E0771-fluc mammary tumors for **(A)** monocytic MDSC (M-MDSCs) (CD45^+^CD11b^+^CD11c^-^I-Ab^-^Ly6C^+^Ly6G^-^) and G-MDSCs (CD45^+^CD11b^+^CD11c^-^I-Ab^-^Ly6C^low^Ly6G^+^). **(B)** Immunogenetic gene expression fold changes of MDSC-related cyto/chemokines in the tumors of HFD- vs LFD-fed animals. **(C)** Concentration of CXCL1 in the tumor microenvironment. Percentage of G-MDSCs expressing **(D)** CXCR2 and **(E)** FasL. **(F)** FasL mean fluorescence intensity of G-MDSCs. **(G)** Percentage of live (annexin V^-^) and apoptotic (annexin V^+^) *in vitro* activated splenic CD8 T cells after 24 h of co-culture with sort-purified tumor-infiltrating G-MDSCs from obese mice in the (top) absence or (bottom) presence of FasL neutralizing antibody. DIO animals were treated with intratumoral anti-Gr-1 or isotype control antibody. Quantification of intratumoral **(H)** MDSCs and **(I)** apoptotic (annexin V^+^) CD8 TILs. Data are pooled from **(A–F, I)** multiple independent experiments or are from **(G, H)** a single representative experiment and presented as means ± SEM. Statistical differences were calculated using parametric t-tests or non-parametric Mann-Whitney U tests as appropriate (*p < 0.05, **p < 0.01, ***p < 0.001, ns = non-significant).

We next sought to identify the mechanism(s) by which obesity promoted MDSC accumulation in murine mammary tumors. Murine MDSCs exist as two main sub-types: monocytic MDSCs (M-MDSCs) and granulocytic MDSCs (G-MDSCs). With obesity, only the G-MDSC subset was increased within tumors (**Figure 3A**). Our tumor immunogenetic profiling results revealed that obesity significantly increased the expression of *Il1b*, *Cxcl1*, *Cxcl3*, *S100a8*, and *Csf3*. These genes encode cytokines and chemokines responsible for the generation and trafficking of G-MDSCs, providing evidence for enhanced MDSC generation and chemotaxis with obesity (**Figure 3B**). Intratumoral protein concentrations of CXCL1 were further found to be elevated in the obese tumor microenvironment (**Figure 3C**). CXCL1 and CXCL3 are chemokines that promote G-MDSC chemotaxis *via* their cognate receptor CXCR2 (25). CXCR2 was highly and equivalently expressed on tumor-infiltrating G-MDSCs from both lean and obese animals (**Figure 3D**). Thus, these results suggest that obesity enhances generation and CXCL1/CXCR2-mediated G-MDSC chemotaxis.

We next asked how obesity impacted FasL expression by G-MDSCs and whether MDSCs were, in fact, responsible for CD8 TIL apoptosis. Approximately 90% of G-MDSCs from lean and DIO animals expressed FasL (**Figure 3E**). Interestingly, although the frequency of G-MDSCs expressing FasL was unchanged, the per cell expression of FasL was elevated with obesity (**Figure 3F**). We next sought to confirm that FasL^+^ G-MDSCs could directly induce Fas/FasL-mediated apoptosis of CD8 T cells. Splenic CD8 T cells were activated *in vitro* to induce expression of Fas then co-cultured with G-MDSCs sort-purified from the tumors of obese mice in the presence or absence of FasL neutralization. FasL neutralization demonstrated that G-MDSCs were not only capable of inducing CD8 T cell apoptosis, but that they did so in a largely FasL-dependent manner (**Figure 3G**). We also asked whether G-MDSC-induced CD8 TIL apoptosis occurred *in vivo*. Short-term anti-Gr-1-mediated MDSC depletion in DIO animals resulted in decreased percentages of apoptotic CD8 TILs relative to isotype-treated controls (**Figures 3H, I**). Thus, these data demonstrate that obesity promotes the recruitment and intratumoral accumulation of FasL^+^ G-MDSCs that functionally suppress hyperactivated Fas^+^ CD8 TILs *via* the induction of apoptosis.

### Obesity Promotes Immunotherapy Resistance and Is Associated With G-MDSC Accumulation

**Figure 4:**
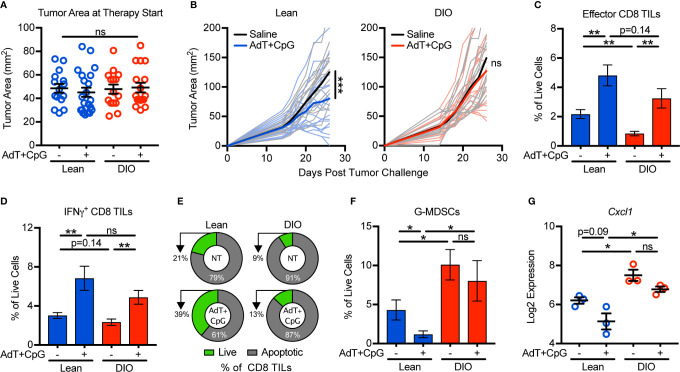
Obesity promotes immunotherapy resistance and is associated with granulocytic myeloid-derived suppressor cell (G-MDSC) accumulation. Lean and DIO mice with established E0771-fluc tumors within a 25–100 mm^2^ range were randomized to receive saline control or AdT+CpG immunotherapy. **(A)** Tumor areas at therapy initiation. **(B)** Resulting tumor growth for lean and DIO animals. Flow cytometric analysis of day 28 excised mammary tumors for **(C)** effector (CD44^+^CD62L^-^) and **(D)** IFNγ^+^ CD8 TILs. **(E)** Percentage of live (annexin V^-^) and apoptotic (annexin V^+^) CD8 TILs. Quantification of **(F)** tumor-infiltrating G-MDSCs (CD45^+^CD11b^+^CD11c^-^I-Ab^-^Ly6C^low^Ly6G^+^). **(G)** Log2 expression of *Cxcl1* in the tumor microenvironment. **(A–D,F)** Data are pooled from multiple independent experiments (n=6–21 per group) or **(E)** are from single a representative experiment (n=4-9 per group) and presented as means ± SEM or **(B)** individual outgrowth is shown, with the bold line indicating mean tumor growth. Statistical differences were calculated using repeated measures two-way ANOVAs with Tukey’s multiple comparison or non-parametric Kruskal-Wallis tests or parametric one-way ANOVAs with *post hoc* pairwise comparison of group means of interest using non-parametric Mann-Whitney U tests, parametric two-tailed unpaired t-tests, or Tukey’s multiple comparisons tests as appropriate (*p < 0.05, **p < 0.01, ***p < 0.001, ns = non-significant).

Our findings that obesity negatively impacted antitumor immunity *via* enhanced G-MDSC accumulation and CD8 TIL apoptosis prompted us to ask if these changes impaired immunotherapy outcomes. Because obesity accelerates tumor growth (**Figure 1**), we first normalized tumor areas at treatment initiation so that tumor burdens were equivalent across groups and thus would not be a confounding variable in comparing outcomes in lean and DIO animals (**Figure 4A**). When tumors were 25–100 mm^2^ in area, lean and DIO animals were randomized in parallel to receive an intratumoral injection of saline or immunotherapy consisting of non-replicative adenovirus (Ad) encoding murine TNF-related apoptosis inducing ligand (TRAIL; AdT) co-administered with CpG oligodeoxynucleotide 1826 (AdT+CpG) (20). AdT induces tumor cell death, increasing tumor antigen availability, while CpG induces antigen-presenting cell activation and maturation (20). Collectively this therapy promotes the priming of endogenous CD8 T cells that subsequently mediate tumor control (20). Additionally, CpG administration induces MDSCs to mature into macrophages and dendritic cells, relieving immunosuppression (26). E0771-fluc cells express both the coxsackievirus and adenovirus receptor (CAR) that permits viral binding and the TRAIL receptor (TRAIL-R2) that induces tumor cell death (**Supplementary Figure 4**). Notably, normalization of tumor burden at immunotherapy administration resulted in equivalent outgrowth of tumors in lean and DIO animals treated with saline (**Figure 4B** direct comparison not shown, p=0.30). In lean mice, AdT+CpG immunotherapy significantly delayed tumor outgrowth, whereas in DIO animals, tumor growth progressed unchecked, demonstrating immunotherapy resistance (**Figure 4B**).

To discern underlying causes of AdT+CpG immunotherapy resistance in obese mice, we examined immune responses after therapy administration. In this setting, both lean and DIO animals had statistically equivalent increases in total effector and IFNγ^+^ CD8 TILs (**Figures 4C, D**). However, following therapy administration, lean mice displayed reductions in CD8 TIL apoptosis and G-MDSC accumulation, whereas neither parameter changed in DIO animals (**Figures 4E, F**). Tumor expression levels of *Cxcl1* followed a similar trend (**Figure 4G**), suggesting that G-MDSC recruitment persisted even after therapy administration in DIO mice. Therefore, although DIO mice mounted CD8 TIL responses equivalent to those of lean animals in response to immunotherapy, high levels of CD8 TIL apoptosis, G-MDSC accumulation, and *Cxcl1* expression remained, corresponding with immunotherapy resistance.

### Disruption of G-MDSC Accumulation Sensitizes Obese Animals to Immunotherapy

**Figure 5:**
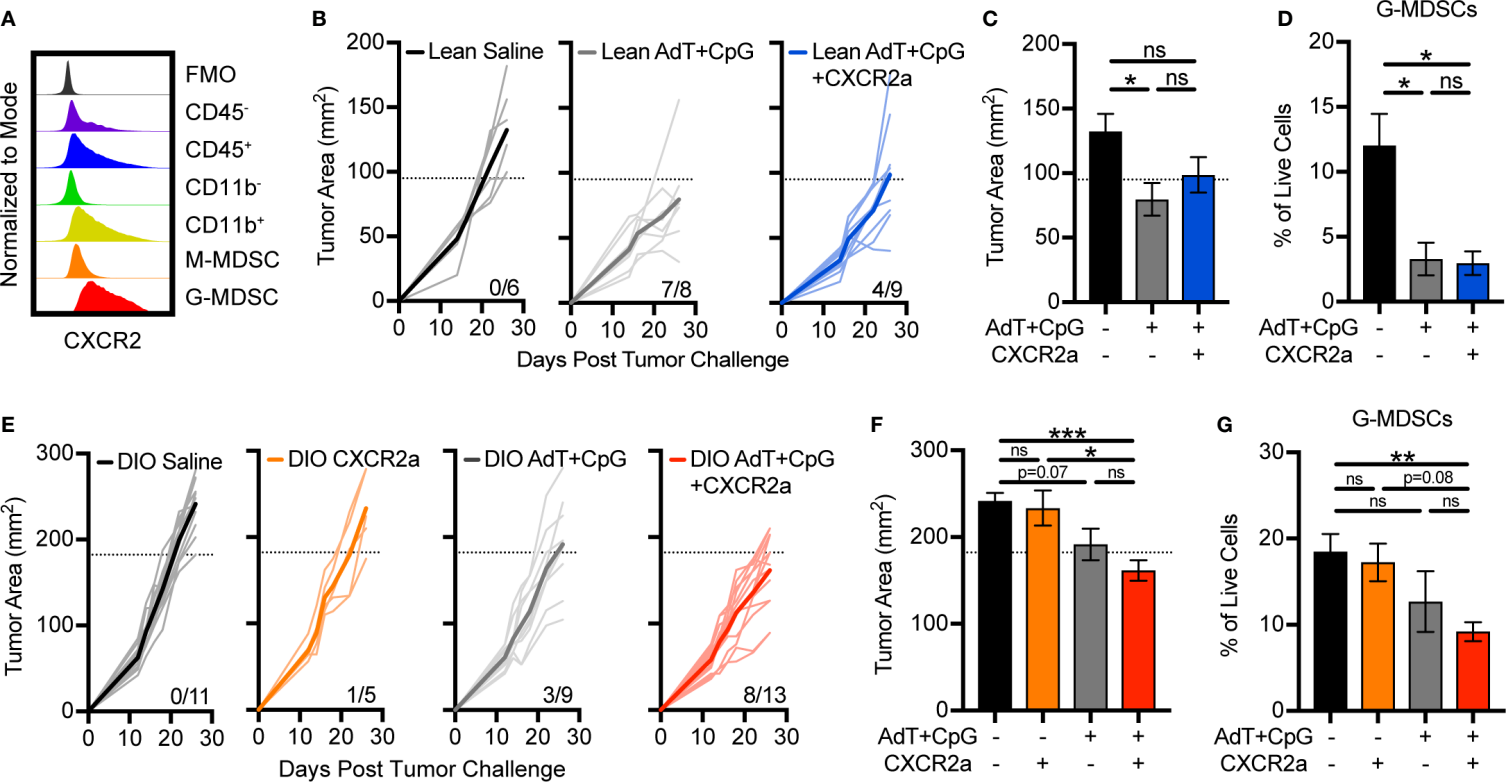
Disruption of granulocytic myeloid-derived suppressor cells (G-MDSC) accumulation sensitizes obese animals to immunotherapy. **(A)** Representative histogram depicting intracellular expression of CXCR2 in different populations in the E0771-fluc tumor microenvironment. Lean and DIO animals bearing E0771-fluc tumors were treated with saline, CXCR2 antagonist (CXCR2a), AdT+CpG, or AdT+CpG+CXCR2a. Resulting tumor growth in **(B)** lean and **(E)** DIO animals is shown. Response thresholds are depicted by the dotted lines which indicate the lower limit of tumor growth in respective saline treated animals. Quantification of **(C, F)** endpoint tumor areas and **(D, G)** tumor-infiltrating G-MDSCs (CD45^+^ CD11b^+^CD11c^-^I-Ab^-^Ly6C^low^Ly6G^+^). Data are pooled from multiple independent experiments and presented as means ± SEM or individual outgrowth is shown, with the bold line indicating mean tumor growth. Statistical differences were calculated using parametric one-way ANOVAs with Tukey’s multiple comparisons tests (*p < 0.05, **p < 0.01, ***p < 0.001, ns = non-significant).

To determine if disruption of G-MDSC accumulation would improve immunotherapy response in lean animals and overcome immunotherapy resistance in obese animals, we targeted CXCR2, which is highly and predominantly expressed on G-MDSCs (**Figures 3D**, **5A**). Treatment of lean animals with combinatorial AdT+CpG+CXCR2 antagonist (CXCR2a) provided no benefit over that of AdT+CpG alone with regard to tumor burden or MDSC accumulation (**Figures 5B–D**). In fact, the combinatorial therapy reduced the proportion of lean animals responding to therapy (7/8 versus 4/9), indicating potential negative effects. Interestingly, no change was seen in G-MDSC accumulation with the addition of the CXCR2a, likely due to the substantial reduction caused by AdT+CpG alone.

Following therapy administration, few beneficial responses were evident in obese animals treated with the CXCR2a (1/5) or AdT+CpG (3/9) alone (**Figure 5E**). However, the combination of the CXCR2a and AdT+CpG led to substantially improved responses in the majority of animals treated (8/13) (**Figure 5E**), corresponding with significant reductions in tumor burdens (**Figure 5F**) and G-MDSC accumulation (**Figure 5G**). Thus, the combinatorial AdT+CpG+CXCR2a immunotherapy was able to reduce G-MDSC accumulation and improve immunotherapy responses, specifically in obese animals.

### CXCL1 Expression in Breast Tumors Is Positively Associated With BMI, Poor Overall Survival, and a MDSC Expression Score

**Figure 6:**
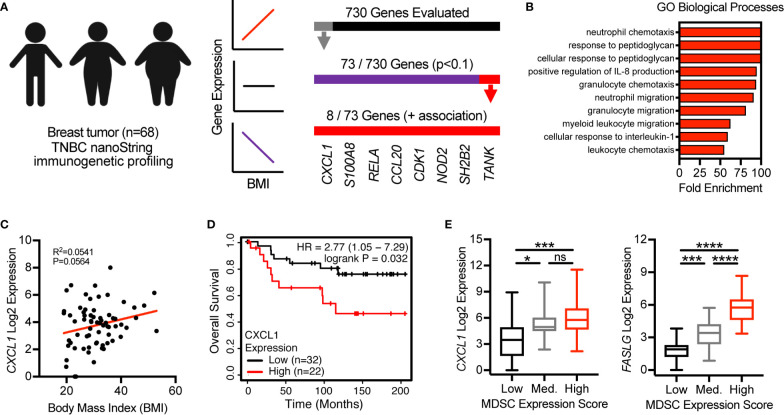
CXCL1 expression in breast tumors is positively associated with BMI, poor overall survival, and a myeloid-derived suppressor cell (MDSC) expression score. **(A)** NanoString immunogenetic profiling of n=68 triple negative breast tumors. Of 730 genes surveyed, 73 were found to be associated (p<0.1) with body mass index (BMI). Of the 73 genes identified, 8/73 were positively associated with BMI and 65/73 were negatively associated with BMI. **(B)** Gene Ontology (GO)-identified biological processes associated with the 8 indicated genes. **(C)** Correlation between BMI and *CXCL1* expression from samples used in **(A**, **B)**. **(D)** Expression of *CXCL1* and overall survival in systemically untreated patients with basal breast cancer. Data were generated using KM Plotter (kmplot.com). **(E)** The Cancer Genome Atlas (TCGA)-derived transcriptomics data from breast tumor tissue of n=88 patients with basal breast cancer. Multi-gene MDSC expression score was calculated and samples were separated into low (n=20), medium (n=20), and high (n=48) tertiles. Analysis of myeloid-derived suppressor cells (MDSC) expression scores versus expression of (left) *CXCL1* and (right) *FASLG*. Data are presented as linear regression, Kaplan-Meier plots, or boxes defining 25^th^ to 75^th^ percentiles with line at median and whiskers extending to minimum and maximum points. Statistical differences were calculated using linear regression, log rank test, or parametric one-way ANOVAs with Tukey’s multiple comparisons tests (*p < 0.05, ***p < 0.001, ****p < 0.0001).

Last, we evaluated the clinical relevance of our findings by utilizing previously published immunogenetic and transcriptomic data from tumor tissues of patients with breast cancer. We first used linear regression analyses to evaluate the association of body mass index (BMI), a metric used to define obesity, with the expression of 730 immune-related genes. In a cohort of 68 patients with triple negative breast cancer (17) 73 of 730 total genes were found to be associated with BMI (p<0.1) (**Figure 6A**). Of these 73 genes, 65 were negatively associated with BMI and 8 were positively associated with BMI. These 8 genes included *CXCL1*, *S100A8*, *RELA*, *CCL20*, *CDK1*, *NOD2*, *SH2B2*, and *TANK* (**Figure 6A**). GO assessment of biological processes associated with these 8 genes revealed neutrophil/granulocyte chemotaxis/migration, corroborating immunogenetic, protein, and cellular results from our murine study (**Figure 6B**). Importantly, *CXCL1*, *S100A8*, and *NOD2* were also found to be positively associated with obesity in our murine study. Increased expression of *CXCL1* was not only found to be associated with increasing BMI (**Figure 6C**), but also decreased overall survival in a cohort of n=54 systemically untreated patients with basal breast cancer (**Figure 6D**). Analysis of transcriptomic data available from The Cancer Genome Atlas (TCGA) allowed us to quantify the relative abundance of MDSCs using a previously published multi-gene MDSC expression score (22). In breast tumor tissues from n=88 patients with basal breast cancer, *CXCL1* and *FASLG* expression were found to increase significantly as the MDSC expression score increased (**Figure 6E**). These data support the clinical relevance of our murine findings by providing evidence that the expression levels of multiple MDSC chemokines (i.e., *CXCL1* and *S100A8*) were positively associated with BMI in patients with breast cancer. High *CXCL1* expression was further associated with poor overall survival and expression-based MDSC abundance. Additionally, this MDSC score was positively associated with expression of *FASLG*.

## Discussion

**Figure 7:**
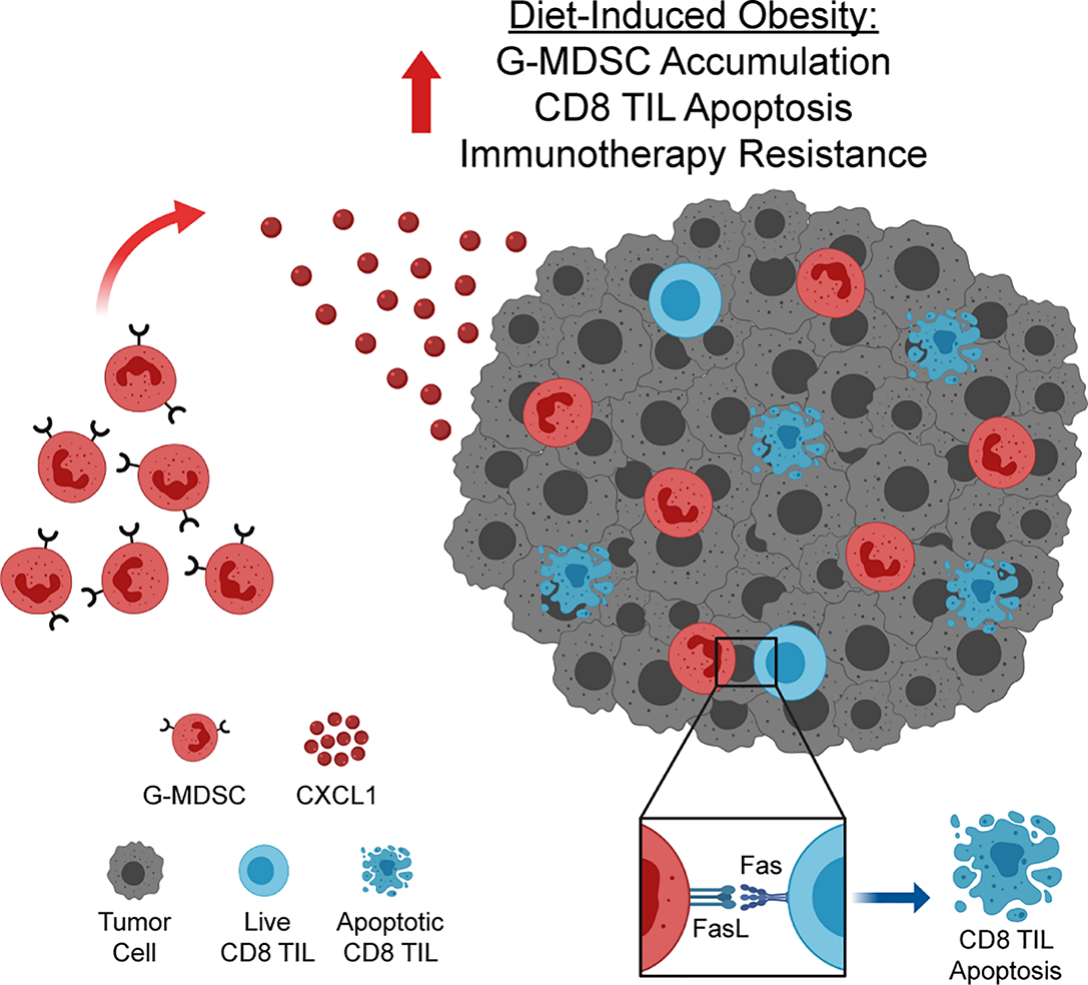
Obesity enhances accumulation of FasL^+^ granulocytic myeloid-derived suppressor cells (G-MDSCs), driving CD8 TIL apoptosis and immunotherapy resistance. Increased concentration of CXCL1 in the obese mammary tumor microenvironment drives CXCL1/CXCR2-mediated accumulation of FasL^+^ G-MDSCs. FasL^+^ G-MDSCs interact with hyperactivated Fas^+^ CD8 TILs, driving CD8 TIL apoptosis and immunotherapy resistance. Graphic created with BioRender.com.

The prevalence of obesity among adults in the US is currently approaching 40% (1) and is expected to reach 50% by 2030 (27). As many cancer patients are now being treated with immunotherapies, understanding the impact of obesity on antitumor immunity and immunotherapeutic outcomes is critical. Here, we report that, in a preclinical model of breast cancer, obesity impairs antitumor immunity and promotes immunotherapy resistance. Specifically, we have identified a novel pathway wherein obesity drives hyperactivation of and subsequent Fas expression on CD8 TILs, priming them for apoptosis. Concurrently, obesity increases intratumoral CXCL1 concentrations, promoting CXCR2-mediated accumulation of FasL^+^ G-MDSCs, resulting in heightened Fas/FasL-mediated CD8 TIL apoptosis and immunotherapy resistance (**Figure 7**). We find evidence of this pathway in lean animals, albeit to a lesser extent (i.e. low levels of both FasL^+^ G-MDSCs and CD8 TIL apoptosis). Thus we posit that the above mechanism is primarily operative in animals with obesity. The clinical relevance of this pathway is illustrated by our transcriptomic data showing high concordant expression of *CXCL1* with increasing BMI, poor survival, and a MDSC expression score in human breast tumors.

Our data add to a growing number of reports detailing the complex role of obesity in modulating antitumor immunity and responses to immunotherapy. Recent reports indicate that obesity not only promotes CD8 T cell dysfunction in a preclinical model of melanoma (28), but also leads to reductions in CD8 TILs in murine and human breast tumors (6). In the latter study, TIL reductions occurred in parallel with increased apoptosis of CD45^+^ leukocytes, although CD8 TIL apoptosis was not specifically examined. We add to this literature by demonstrating that obesity not only drives hyperactivation of CD8 TILs, but also predisposes them to G-MDSC-mediated apoptosis, thereby impeding antitumor immunity and immunotherapeutic efficacy (29).

Obesity and inflammation expand and mobilize MDSCs and their precursors *via* molecules such as IL-6, CRP, IL-1β, and leptin (13–16, 30), increasing their susceptibility to tumor-derived signals that further drive their recruitment and suppressive capacity. Here, we found that the expression levels of multiple mediators of MDSC development and chemotaxis, including *Cxcl1*, *Cxcl3*, *S100a8*, *Csf3* (G-CSF), and *Il1β* (31), were elevated in the tumors of DIO animals (**Figures 1E**, **3B** and **Supplementary Table 1**). These results were further supported by our clinical analyses which demonstrated positive associations between BMI and the expression of *CXCL1* and *S100A8* in human breast tumors (**Figure 6**). These data provide evidence for redundant mechanisms of MDSC recruitment to the tumor microenvironment in mice and humans with obesity, highlighting the need for further investigation of obesity-associated alterations in the soluble protein milieu of the tumor microenvironment. Here, we targeted CXCR2 for intervention in mice with mammary tumors, disrupting signals from its cognate ligands, CXCL1 and CXCL3. Although a significant therapeutic benefit was observed, chemotactic redundancy (i.e., S100A8) was likely responsible for achieving only a modest reduction in tumor burden. This redundancy may serve as a barrier for the clinical targeting of MDSCs, particularly in the context of obesity. Thus, interventions targeting MDSC generation or function, rather than chemotaxis, may be more efficacious in the clinical setting.

A recent study utilized single cell transcriptomics and the MMTV-PyMT transgenic murine model of breast cancer to identify expression signatures found in G-MDSCs versus their non-suppressive neutrophil counterparts (32). The authors identified genes, including *Arg2*, *Cxcr2*, *Il1b*, *Ifitm1*, *Socs3*, *Osm*, and *Cd14* among others, whose expression was elevated in G-MDSCs. These genes identify particular mechanisms of suppression (i.e., *Arg2*, which encodes arginase 2) and chemotaxis (i.e., *Cxcr2*) utilized by G-MDSCs (25, 33). In our study, all of the aforementioned genes were increased in expression in the tumors of obese mice (**Supplementary Table 2**). These data further support the classification of G-MDSCs in our murine model and emphasize the accumulation of G-MDSCs rather than non-suppressive neutrophils.

Recently, tumor-infiltrating MDSCs have been shown to promote immunotherapy resistance in preclinical models of breast cancer and melanoma (17, 18). In studies of inducible and transplantable melanoma, MDSC-mediated immunotherapy resistance was found to occur in part through expression of FasL and subsequent Fas/FasL-mediated apoptosis of CD8 TILs (18). Here, we substantiated those findings and extended them to breast cancer, where we implicate FasL-expressing MDSCs as part of a broader pathway that links host obesity to heightened CD8 TIL apoptosis and immunotherapy resistance. We find that targeting of MDSCs is sufficient to improve immunotherapy response, although the subsequent impact on CD8 TIL dynamics was not evaluated. Obesity is an incredibly complex and multifactorial disease that elicits numerous changes to the breast tumor microenvironment (34). As such, we recognize the potential for additional known, or currently unknown, factors to contribute to our observed outcomes.

Reports indicate that elevated levels of plasma IL-6 and CRP are associated with reduced progression free and overall survival in patients with breast cancer who were treated with anti-PD-L1 immunotherapy (12). Notably, both IL-6 and CRP are elevated with obesity and modulate MDSC dynamics (15, 16). Although the impact of obesity on immunotherapy outcomes in patients with breast cancer has not yet been evaluated, the aforementioned data suggest that obesity may be associated with worse clinical outcomes in breast cancer, which may be linked to MDSC accumulation. This notion is backed by our transcriptomic analyses, which shows a positive correlation between *CXCL1* expression and BMI, poor survival, and a MDSC expression score in human breast tumor tissues (**Figure 4E**). Additional evidence for this hypothesis comes from earlier work implicating obesity in resistance to targeted and chemotherapies in patients with breast cancer (7, 8). Our preclinical findings concur with these outcomes. Conversely, a retrospective analysis of women with melanoma found that obesity did not impact outcomes to anti-PD-1/PD-L1 immunotherapy (35). As this study was retrospective, there was no evaluation of IL-6, CRP, or MDSCs, indicating areas for future prospective analyses. Thus, these data suggest obesity may be associated with worse immunotherapy outcomes in patients with breast cancer, but also that obesity may differentially impact treatment outcomes based on tumor or immunotherapy type, emphasizing the need for continued study of this critical issue.

With nearly 40% of the adult US population presenting with obesity and an additional 30% presenting with overweight (1), it is critical now more than ever that we consider co-morbidities, such as obesity and diabetes, as the new normal. This is especially needed in preclinical modeling where the use of young lean mice starkly departs from our aging and weighty cancer patient population. Forethought will allow us to more considerately and effectively develop therapeutics to treat what continues to be the increasing majority of our population.

## Data Availability Statement

The differentially expressed genes identified from the murine mammary tumor immunogenetic profiling results in this study can be found in **Supplementary Table 2**. Immunogenetic and transcriptomic profiling data from human breast tumors were obtained from a previously published manuscript (17) or TCGA and are publicly available.

## Ethics Statement

All animal experiments were conducted as approved by the Animal Resources Program and Institutional Animal Care and Use Committee at the University of Alabama at Birmingham (UAB).

## Author Contributions

JG and LN conceptualized the study, designed experiments, and wrote the manuscript. JG managed and led the study under the supervision of LN. JG, RO, WT, and MB performed experiments and analyzed data. All authors contributed to the article and approved the submitted version.

## Funding

Research reported in this publication was supported by the University of Alabama at Birmingham Department of Nutrition Sciences startup package to LN, Breast Cancer Research Foundation of Alabama Impact Grant to LN, NIH award #T32GM008111 to JG, NCI award #T32CA047888 to RO and MB, NCI award #T32CA183926 to WT, the Rita Allen Foundation grant #2014701 to RS, and NIH awards #P30CA013148 and #1S10OD021697 to the UAB O’Neal Comprehensive Cancer Center Preclinical Imaging Shared Facility.

## Conflict of Interest

The authors declare that the research was conducted in the absence of any commercial or financial relationships that could be construed as a potential conflict of interest.
