# Supplementary material for: Obesity-Associated Myeloid-Derived Suppressor Cells Promote Apoptosis of Tumor-Infiltrating CD8 T Cells and Immunotherapy Resistance in Breast Cancer
Source: Front Immunol. 2020 Oct 6;11:590794. doi: 10.3389/fimmu.2020.590794 (PMC7573510; doi:10.3389/fimmu.2020.590794)
Supplement: Supplementary file 1 [file Table_1.docx]

Supplementary Material

# Supplementary Figures


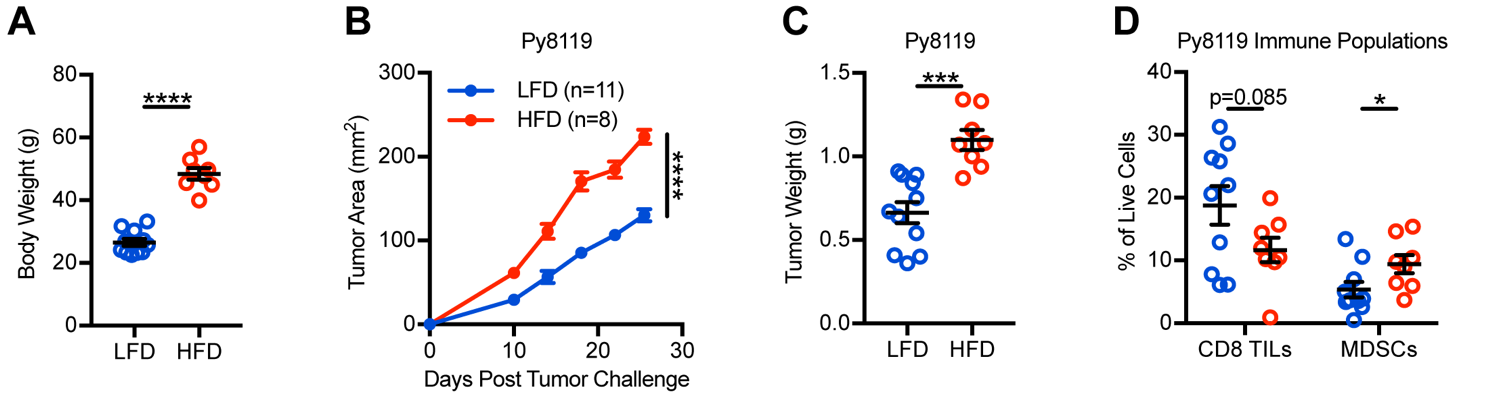


**Supplementary Figure 1. Diet-induced obesity promotes Py8119 mammary tumor growth and remodeling of the tumor immune microenvironment.** (**A**) Body weight of animals 16 weeks after randomization to low-fat diet (LFD; 14% kcal from fat) or high-fat diet (HFD; 60% kcal from fat). Following diet administration, animals were tumor challenged with the Py8119 murine mammary tumor cell line and tumor growth was monitored via (**B**) caliper measurements and (**C**) excised tumor weights. (**D**) Flow cytometric analysis of day 24-25 excised mammary tumors for tumor infiltrating CD3^+^CD8^+^ TILs and CD45^+^CD11b^+^CD11c^-^I-Ab^-^Ly6C^+^Ly6G^+^ MDSCs. Data are pooled from multiple independent experiments and presented as means ± SEM. Statistical differences were calculated using parametric t-tests or non-parametric Mann-Whitney U tests as appropriate and repeated measures two-way ANOVA with Tukey’s multiple comparisons tests (*p<0.05, ***p<0.001, ****p<0.0001).


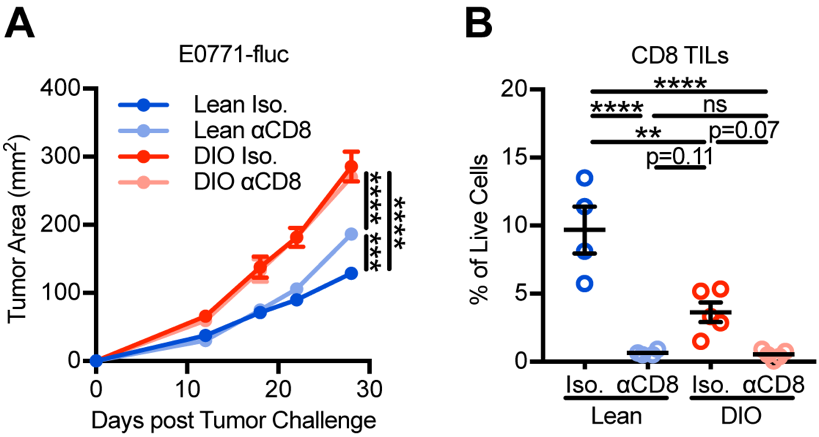


**Supplementary Figure S2. Reduced CD8 TIL abundance with obesity is associated with impaired anti-tumor immunity.** Following diet randomization, LFD-fed lean and HFD-fed DIO animals were challenged with E0771-fluc mammary tumor cells. On days 1, 3, 5, 7, 14, 21, and 27 following tumor challenge, animals received intraperitoneal injections of 100ug of isotype control antibody or anti-CD8 depletion antibody. (**A**) Tumor growth in lean and DIO animals receiving isotype control or anti-CD8 depletion antibodies and (**B**) levels of CD8 TILs present at day 28 endpoint. Data are from a single experiment where n=4-5 per group and presented as average tumor growth or means ± SEM. Statistical differences were calculated using parametric repeated measures two-way ANOVA or one-way ANOVA as appropriate, both with Tukey’s multiple comparisons tests (**p<0.01, ***p<0.001, ****p<0.0001, ns=non-significant).


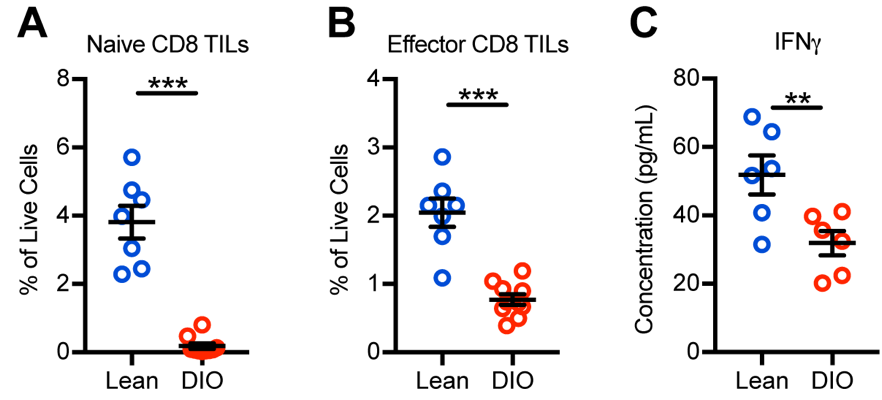


**Supplementary Figure S3. Obesity is associated with reduced percentages of naive and effector CD8 TILs and total IFNγ.** Flow cytometric analysis of day 28 excised E0771-fluc mammary tumors for (**A**) naive (CD44^-^CD62L^+^) and (**B**) effector (CD44^+^CD62L^-^) CD8^+^ tumor infiltrating lymphocytes (TILs). (**C**) Concentration of IFNγ in the tumor microenvironment. Data are pooled from multiple independent experiments and presented as means ± SEM. Statistical differences were calculated using parametric t-tests or non-parametric Mann-Whitney U tests as appropriate (**p<0.01, ***p<0.001, ns=non-significant).


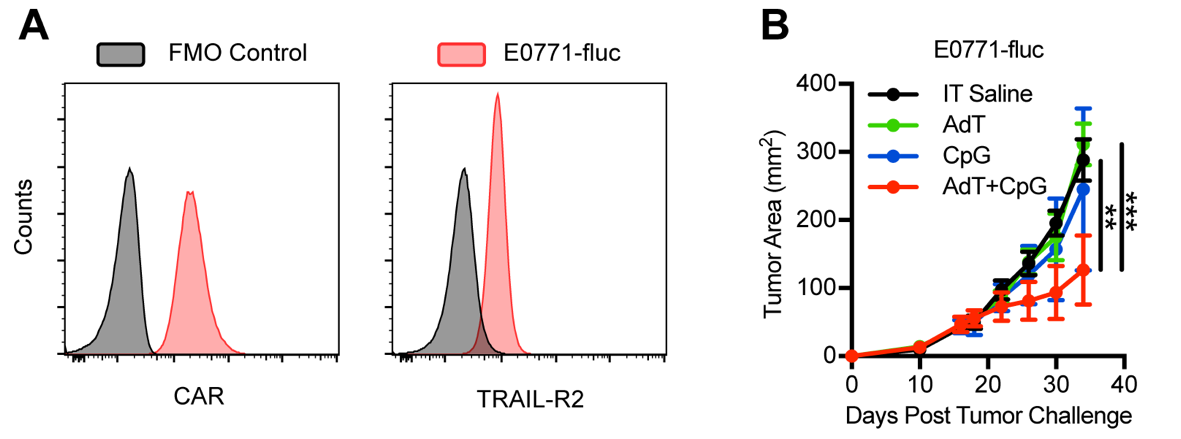


**Supplementary Figure 4. Combinatorial AdT+CpG immunotherapy reduces growth of E0771-fluc mammary tumors.** (**A**) *In vitro* E0771-fluc expression of coxsackievirus and adenovirus receptor (CAR) and TRAIL receptor 2 (TRAILR2) which allow for adenovirus binding and subsequent expression of TRAIL, leading to TRAIL-R2-mediated tumor cell death. (**B**) E0771-fluc tumor growth for 10-12 week old lean mice receiving intratumoral injections of saline control, AdT, CpG, or AdT+CpG when established tumors were 25-100mm^2^ in size. Data are from a single experiment where n=3/group and presented as means ± SEM. Statistical differences were calculated using repeated measures two-way ANOVA with Tukey’s multiple comparisons test (**p<0.01, ***p<0.001).

# Supplementary Tables

**Supplementary Table 1. List of antibodies and key reagents used in this study.**

| **Antibodies** | | | | | |
| --- | --- | --- | --- | --- | --- |
| **Target** | **Clone** | **Vendor** | **Catalog Number** | **Fluorophore** | **RRID** |
| Bcl-2 | BCL/10C4 | BioLegend | 633510 | A647 | AB_2274702 |
| CAR | RmcB | MilliporeSigma | 05-644 | Unconjugated | AB_309871 |
| CD3 | 17A2 | BioLegend | 100236 | APC | AB_2561456 |
| CD3 | 17A2 | BioLegend | 100217 | PerCP/Cy5.5 | AB_1595597 |
| CD3e | 145-2C11 | BioLegend | 100351 | BV605 | AB_2565842 |
| CD8a | 53-6.7 | BioLegend | 100723 | A488 | AB_389304 |
| CD11b | M1/70 | BioLegend | 101216 | PE/Cy7 | AB_312799 |
| CD11c | N418 | BioLegend | 117324 | APC/Cy7 | AB_830649 |
| CD19 | 6D5 | BioLegend | 115539 | BV605 | AB_11203538 |
| CD44 | IM7 | BioLegend | 103032 | PerCP/Cy5.5 | AB_2076204 |
| CD45 | 30-F11 | BioLegend | 103126 | Pac Blue | AB_493535 |
| CD45 | 30-F11 | BioLegend | 103116 | APC/Cy7 | AB_312981 |
| CD62L | MEL-14 | BioLegend | 104417 | PE/Cy7 | AB_313102 |
| CD95 (Fas) | SA367H8 | BioLegend | 152603 | APC | AB_2632898 |
| CD178 (FasL) | MFL3 | BioLegend | 106603 | Biotin | AB_313276 |
| CD279 (PD-1) | 29F.1A12 | BioLegend | 135218 | BV421 | AB_2561447 |
| CD182 (CXCR2) | SA044G4 | BioLegend | 149304 | PE | AB_2565692 |
| Gr-1 (Ly-6C/Ly-6G) | RB6-8C5 | BioLegend | 108423 | APC/Cy7 | AB_2137486 |
| I-Ab (MHC-II) | KH74 | BioLegend | 115307 | A488 | AB_493138 |
| IFN-γ | XMG1.2 | BioLegend | 505830 | BV421 | AB_2563105 |
| Ki-67 | 16A8 | BioLegend | 652404 | PE | AB_2561525 |
| Ly6C | HK1.4 | BioLegend | 128036 | BV605 | AB_2562353 |
| Ly6G | 1A8 | BioLegend | 127615 | PerCP/Cy5.5 | AB_1877272 |
| mIgG | - | Fisher | F-2761 | FITC | AB_2536524 |
| TRAIL-R2 (CD262) | MD5-1 | BioLegend | 119905 | PE | AB_345401 |
| ***In vitro* and *in vivo* antibodies** | | | | | |
| **Target** | **Clone** | **Vendor** | **Catalog Number** | **Fluorophore** | **RRID** |
| CD8β | 53-5.8 | Bio X Cell | BE0223 | Unconjugated | AB_2687706 |
| Rat IgG1 Isotype Control | TNP6A7 | Bio X Cell | BE0290 | Unconjugated | AB_2687813 |
| CD178 (FasL) | MFL3 | Bio X Cell | BE0319 | Unconjugated | AB_2819046 |
| Gr-1 (Ly-6C/Ly-6G) | RB6-8C5 | Bio X Cell | BP0075 | Unconjugated | AB_10312146 |
| Rat IgG2b Isotype Control | LTF-2 | Bio X Cell | BP0090 | Unconjugated | AB_1107780 |
| **Reagents** | | | | | |
| **Item** | | **Vendor** | | **Catalog Number** | |
| Zombie Aqua Fixable Viability Kit | | BioLegend | | 423102 | |
| Streptavidin-PE | | BioLegend | | 405203 | |
| Annexin V Apoptosis Detection Kit | | Fisher | | BDB559763 | |
| SB 225002 (CXCR2 antagonist) | | Fisher | | 27-255-0 | |

**Supplementary Table 2. List of DE genes from Figure 1 (DIO vs lean).**

| **Downregulated** | | | |  | **Upregulated** | | | |
| --- | --- | --- | --- | --- | --- | --- | --- | --- |
| **Gene** | **Log2 fold change** | **Fold Change** | **P-value** |  | **Gene** | **Log2 fold change** | **Fold Change** | **P-value** |
| *Psmb9* | -0.582 | -1.50 | 0.00285 |  | *Csf3* | 3.61 | 12.21 | 0.00425 |
| *Pik3cd* | -0.611 | -1.53 | 0.039 |  | *Chit1* | 3.27 | 9.65 | 0.0353 |
| *Ikzf2* | -0.631 | -1.55 | 0.0192 |  | *S100a8* | 2.54 | 5.82 | 0.0145 |
| *Stat1* | -0.654 | -1.57 | 0.0114 |  | *Nfatc4* | 2.37 | 5.17 | 0.000111 |
| *Il15ra* | -0.654 | -1.57 | 0.0133 |  | *Ifitm1* | 2.32 | 4.99 | 0.00847 |
| *Irf1* | -0.682 | -1.60 | 0.00317 |  | *Lcn2* | 2.19 | 4.56 | 0.00837 |
| *Irgm2* | -0.712 | -1.64 | 0.0323 |  | *C8b* | 2.09 | 4.26 | 0.0397 |
| *Gzma* | -0.75 | -1.68 | 0.0496 |  | *Cxcr2* | 2.05 | 4.14 | 0.00475 |
| *Tnfrsf14* | -0.832 | -1.78 | 0.0207 |  | *Cd14* | 1.95 | 3.86 | 0.00588 |
| *C1ra* | -0.852 | -1.81 | 0.0197 |  | *Cxcl3* | 1.91 | 3.76 | 0.0177 |
| *Tmem173* | -0.922 | -1.89 | 0.00342 |  | *Il1a* | 1.79 | 3.46 | 0.0425 |
| *Icos* | -0.936 | -1.91 | 0.0157 |  | *Arg2* | 1.75 | 3.36 | 0.0211 |
| *Ctsw* | -1.05 | -2.07 | 0.0421 |  | *Cxcl1* | 1.38 | 2.60 | 0.00126 |
| *Cd83* | -1.11 | -2.16 | 0.0204 |  | *Mmp9* | 1.34 | 2.53 | 0.0268 |
| *Lck* | -1.19 | -2.28 | 0.00955 |  | *Nod2* | 1.1 | 2.14 | 0.00842 |
| *Il21r* | -1.21 | -2.31 | 0.0377 |  | *Tnfsf12* | 1.03 | 2.04 | 0.00437 |
| *Klrk1* | -1.25 | -2.38 | 0.0202 |  | *Chil3* | 0.986 | 1.98 | 0.0296 |
| *H2-DMa* | -1.25 | -2.38 | 0.0357 |  | *Osm* | 0.927 | 1.90 | 0.0451 |
| *Klrc1* | -1.31 | -2.48 | 0.0221 |  | *Il1b* | 0.831 | 1.78 | 0.0484 |
| *Ccr7* | -1.32 | -2.50 | 0.0124 |  | *Socs3* | 0.804 | 1.75 | 0.0113 |
| *Cxcr6* | -1.35 | -2.55 | 0.0271 |  | *Mapk3* | 0.643 | 1.56 | 0.00214 |
| *Il2rb* | -1.37 | -2.58 | 4.38E-05 |  | *Cebpb* | 0.612 | 1.53 | 0.0426 |
| *H2-Ab1* | -1.42 | -2.68 | 0.0179 |  |  |  |  |  |
| *Il24* | -1.43 | -2.69 | 0.0388 |  |  |  |  |  |
| *Tnfsf15* | -1.45 | -2.73 | 0.0372 |  |  |  |  |  |
| *Cxcl9* | -1.5 | -2.83 | 0.0127 |  |  |  |  |  |
| *Cd22* | -1.5 | -2.83 | 0.0248 |  |  |  |  |  |
| *Ncr1* | -1.57 | -2.97 | 0.0442 |  |  |  |  |  |
| *Klrd1* | -1.58 | -2.99 | 0.00218 |  |  |  |  |  |
| *Cd74* | -1.59 | -3.01 | 0.00755 |  |  |  |  |  |
| *Pin1* | -1.62 | -3.07 | 0.0375 |  |  |  |  |  |
| *Tnfrsf4* | -1.67 | -3.18 | 0.0385 |  |  |  |  |  |
| *Klra7* | -1.68 | -3.20 | 0.00989 |  |  |  |  |  |
| *Cd3g* | -1.68 | -3.20 | 0.013 |  |  |  |  |  |
| *Card11* | -1.69 | -3.23 | 0.0102 |  |  |  |  |  |
| *Il5* | -1.7 | -3.25 | 0.0115 |  |  |  |  |  |
| *H2-DMb1* | -1.72 | -3.29 | 0.000329 |  |  |  |  |  |
| *Il7* | -1.73 | -3.32 | 0.0369 |  |  |  |  |  |
| *Slamf6* | -1.77 | -3.41 | 0.022 |  |  |  |  |  |
| *H2-Aa* | -1.78 | -3.43 | 0.00708 |  |  |  |  |  |
| *Klrb1c* | -1.79 | -3.46 | 0.0127 |  |  |  |  |  |
| *H2-Eb1* | -1.81 | -3.51 | 0.00639 |  |  |  |  |  |
| *Ccl1* | -1.92 | -3.78 | 0.0225 |  |  |  |  |  |
| *Cxcr3* | -2 | -4.00 | 0.0121 |  |  |  |  |  |
| *Cx3cl1* | -2.05 | -4.14 | 0.00793 |  |  |  |  |  |
| *Xcl1* | -2.09 | -4.26 | 0.00496 |  |  |  |  |  |
| *Itk* | -2.14 | -4.41 | 0.00552 |  |  |  |  |  |
| *H2-DMb2* | -2.15 | -4.44 | 0.0399 |  |  |  |  |  |
| *Bcl2* | -2.17 | -4.50 | 0.046 |  |  |  |  |  |
| *Pdcd1lg2* | -2.21 | -4.63 | 0.0058 |  |  |  |  |  |
| *Cd8a* | -2.22 | -4.66 | 0.0184 |  |  |  |  |  |
| *Itgae* | -2.23 | -4.69 | 0.0063 |  |  |  |  |  |
| *Tnfsf13b* | -2.32 | -4.99 | 0.041 |  |  |  |  |  |
| *Cd6* | -2.32 | -4.99 | 0.0471 |  |  |  |  |  |
| *Cx3cr1* | -2.39 | -5.24 | 0.00163 |  |  |  |  |  |
| *Zap70* | -2.42 | -5.35 | 0.0474 |  |  |  |  |  |
| *Ccr8* | -2.43 | -5.39 | 0.00321 |  |  |  |  |  |
| *H2-Ob* | -2.58 | -5.98 | 0.0161 |  |  |  |  |  |
| *Il12b* | -2.68 | -6.41 | 0.0229 |  |  |  |  |  |
| *Runx3* | -2.81 | -7.01 | 0.0231 |  |  |  |  |  |
| *Timd4* | -3.58 | -11.96 | 0.0119 |  |  |  |  |  |
| *Gbp2b* | -3.77 | -13.64 | 0.0118 |  |  |  |  |  |
| *Cd79b* | -3.87 | -14.62 | 0.0156 |  |  |  |  |  |
| *Ms4a1* | -4.72 | -26.35 | 0.00899 |  |  |  |  |  |
